# Magnetoimpedance and Stress-Impedance Effects in Amorphous CoFeSiB Ribbons at Elevated Temperatures

**DOI:** 10.3390/ma13143216

**Published:** 2020-07-19

**Authors:** Dmitriy A. Bukreev, Michael S. Derevyanko, Alexey A. Moiseev, Alexander V. Semirov, Peter A. Savin, Galina V. Kurlyandskaya

**Affiliations:** 1 Department of Physics, Irkutsk State University, 1 Karl Marx St., 664003 Irkutsk, Russia; da.bukreev@gmail.com (D.A.B.); mr.derevyanko@gmail.com (M.S.D.); moiseev.al.an@gmail.com (A.A.M.); semirov@mail.ru (A.V.S.); 2 Department of Magnetism and Magnetic Nanomaterials, INSM, Ural Federal University, 19 Mira St., 620002 Ekaterinburg, Russia; Peter.Savin@urfu.ru; 3 Departamento de Electricidad y Electrónica, Universidad del País Vasco UPV-EHU and BCMaterials, 48940 Leoa, Spain

**Keywords:** magnetoimpedance, stress impedance, sensors, thermal stability of sensor response, soft magnetic amorphous alloys, magnetostriction

## Abstract

The temperature dependencies of magnetoimpedance (MI) and stress impedance (SI) were analyzed both in the as-quenched soft magnetic Co_68.5_Fe_4_Si_15_B_12.5_ ribbons and after their heat treatment at 425 K for 8 h. It was found that MI shows weak changes under the influence of mechanical stresses in the temperature range of 295–325 K and SI does not exceed 10%. At higher temperatures, the MI changes significantly under the influence of mechanical stresses, and SI variations reach 30%. Changes in the magnetoelastic properties for the different temperatures were taken into consideration for the discussion of the observed MI and SI responses. The solutions for the problem of thermal stability of the magnetic sensors working on the principles of MI or SI were discussed taking into account the joint contributions of the temperature and the applied mechanical stresses.

## 1. Introduction

There are different sensing technologies based on the coupling of the magnetic and electric/ elastic properties of soft ferromagnets [1,2]. The magnetoelastic resonance of amorphous ribbons was proven to be capable to ensure the precise measurements of the viscosity of technologically important fluids, such as lubricant oils [3] or the properties of biological samples [4]. High-frequency electrical properties of amorphous soft magnetic alloys are strongly sensitive to various external effects causing a change of the magnetic permeability [5]. In particular, the magnetoimpedance (MI) [6,7,8] and the stress-impedance (SI) [9,10] effects, consisting in a change of the total electric impedance of a ferromagnetic conductor under the influence of the external magnetic field and deformations, respectively, are well studied phenomena in amorphous and nanocrystalline wires, composite wires [11], ribbons and thin films. In some cases, they were investigated in a condition of application of torsional stress [12].

The MI and SI are very promising for the creation of highly-sensitive detectors of various external physical parameters [13,14,15,16] that can be appropriate for different kinds of applications including biology and medicine [17,18,19]. Therefore, despite a rather long history of MI and SI effect investigation, the fundamentals related to these phenomena and the search for new MI and SI materials are still under the special attention of researchers.

MI sensors for many applications require enhanced thermal stability in the working temperature range. Therefore, it is necessary to investigate the temperature dependence of MI responses and their temperature stability [20,21]. It should be noted that MI sensitive elements very often consist of different kind of materials [16,17], having different electrical conductivity values and different thermal expansion coefficients. Therefore, a change in the temperature can result in the appearance or modification of the distribution of mechanical stresses in the MI element and change the output signal [22]. For example, it was found that the temperature change in the MI of the elastically deformed Co-based amorphous ribbon can reach 3%/K, while in the absence of deformation the temperature changes do not exceed the value of 0.5%/K [23]. Thereby, it is not sufficient to take into account the contribution of the temperature for the development of the thermostable MI sensors with a high range of functional temperatures. In this case, the investigation of the influence of both the temperature and mechanical stresses in the formation of the MI responses is necessary.

From s fundamental point of view, these investigations allow to study the temperature changes in the magnetoelastic properties of the amorphous soft magnetic alloys. It is important because a magnetic anisotropy of the amorphous soft magnetic alloys has mainly a magnetoelastic nature [3,24]. For example, the investigation of the temperature dependence of the impedance of the elastically deformed Co-based ribbons [23] and wires [25] showed that a magnetostriction sign can be changed and compensation magnetostriction temperature can be determined.

In this work, the temperature dependencies of MI and SI effect observed in Co-based amorphous ribbons were studied in a view of the MI sensors’ thermal stability increase that was discussed for wide ranges of alternating current frequencies.

## 2. Materials and Methods

### 2.1. Samples

The amorphous ribbons with a thickness of 24 μm and a width of 710 μm (nominal composition Co_68.5_Fe_4_Si_15_B_12.5_) were prepared using a rapid quenching technique onto the surface of the Cu weal.

Co-rich amorphous wires and ribbons are well known due to their excellent magnetoimpedance properties related to the extra magnetic softness closely connected to low magnetoelastic anisotropy. Co_68.5_Fe_4_Si_15_B_12.5_ composition amorphous ribbons are very convenient materials as they have quite a high Curie point [26] of about 630 K, allowing the temperature dependence of the magnetoimpedance investigation in the practically important range of technological temperatures. In addition, this particular composition has such a technological advantage as the possibility of high-level surface properties’ control. The idea to use an amorphous ribbon-based GMI (giant magnetoimpedance effect) biosensor for both magnetic label and label-free detection was proposed long ago and it is currently under active development [27,28]. The quality of the surface of sensitive elements is crucial for biosensing purposes [29].

Magnetic hysteresis loops were obtained by the induction method in a longitudinal magnetic field (applied along the long side of the rectangular elongated sample) with a frequency of 1 kHz. The magnetic field amplitude was as high as 1.5 kA/m. The saturation magnetization (*M_S_*) at room temperature was as high as *M_S_* = 560 MA/m, the coercive force *H_C_* ≈ 50 A/m and the Curie temperature *T_C_* = 630 K.

The ribbons of 30 mm length were used for the magnetoimpedance and stress-impedance investigation. The samples were studied both in as-quenched state (S-AQ) and after the heat treatment (S-HT). The thermal treatment was carried out at the temperature of 425 K for 8 h.

### 2.2. The Impedance Measurements

**Figure 1:**
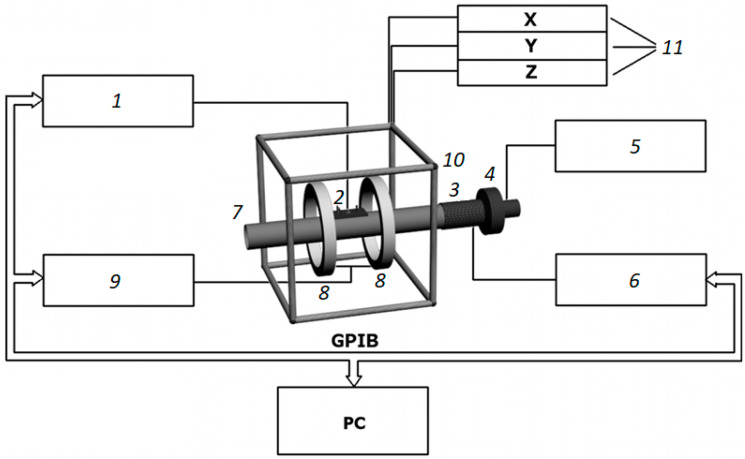
Scheme of THE experimental setup. 1—impedance analyzer; 2—measuring cell; 3—heater; 4—air blower; 5—power source for the air blower; 6—power source of the heater; 7—duct; 8—Helmholtz coils; 9—power source for the Helmholtz coils; 10—magnetic coils of the compensation system; 11—power sources of the compensation system; GPIB— General Purpose Interface Bus; PC—personal computer.

**Figure 2:**
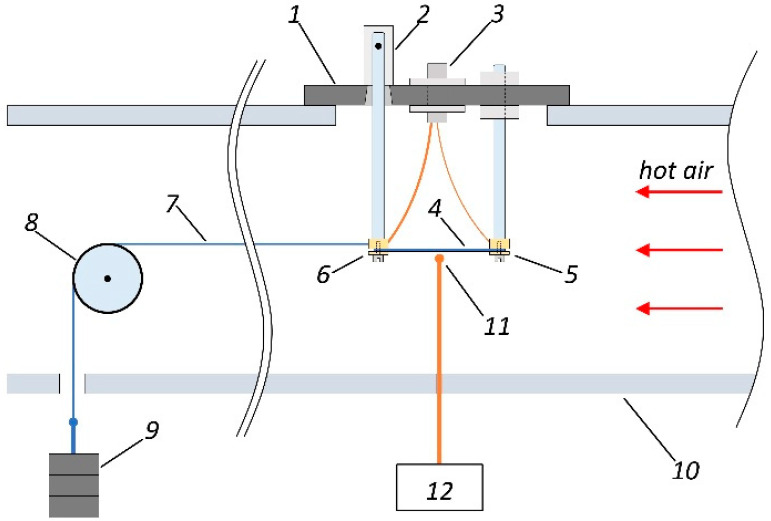
Scheme of the measuring cell placement in the duct. 1—measuring cell base; 2—swivel of the movable contact and the base; 3—SMA connector; 4—sample; 5—fixed contact; 6—movable contact; 7—Kevlar thread; 8—block; 9—stacked load; 10—air duct; 11—thermocouple; 12—digital millivoltmeter.

The impedance was measured using a homemade automatic setup. It allowed to investigate the simultaneous contributions of the magnetic field, mechanical stresses, and temperature on the impedance of ferromagnetic conductors with different geometries, including the geometry of amorphous ribbons. The Agilent 4294A impedance analyzer is a main part of the setup (Figure 1). The Impedance Probe 42941A (Keysight Technologies, Santa Rosa, CA, USA) is used in order to connect the analyzer with the measuring cell. The possibility to compensate the contribution of self-impedance of the measuring cell is an important part of the measurements, which was always used for system calibration. In addition, the measuring system included a thermocouple connected with a millivoltmeter (Figure 2). It should be noted that a thermocouple was situated in close proximity but not in direct contact with the ribbon surface, in order to exclude a distortion of the measuring results. We made calibration tables for the whole of the temperature range that allowed to determine a sample’s temperature using a direct datum of flow temperature.

The external magnetic field was created by the pair of Helmholtz coils. They were connected to a power supply, ensuring a maximum magnetic field value of ±12.5 kA/m. Three pairs of orthogonal magnetic field coils connected to three independent stabilized power supplies were used for the careful compensation of geomagnetic and effective laboratory fields (Figure 1). The sample was heated by the air stream (or argon gas). The maximum possible temperature was as high as 775 K. The measuring cell was mounted on the air duct as shown in Figure 2.

The base of the measuring cell was made of a heat-resistant dielectric material. The sample was attached to the contacts as shown in Figure 2. The contacts were silver plated aiming to avoid oxidation during heating. One of the contacts was fixed on the base rigidly. The second contact was mobile, because it has a swivel connection with the base of the cell. First, this provided a free change in the length of the sample with temperature. Secondly, this construction allowed the application of the force to the sample for creating external tensile stresses. An SMA (SubMiniature version A) connector (Tyco Electronics Ltd., Schaffhausen, Switzerland) was used for the electric connection with the contacts. The Impedance Probe 42941A was connected to this jack.

A Kevlar thread was attached to the movable contact of the measuring cell in order to create tensile stresses in the sample. Another end of the thread was connected to the stacked load as shown in Figure 2.

The typical Young’s modulus, *E*, for the Co-based amorphous alloys, is about 200 GPa [30,31]. According to Hooke’s law, it can be determined that the maximum elongation of the sample is approximately 1 × 10^−4^ m at *σ_max_* = 690 MPa (corresponding to the maximum value of the mechanical stresses in this study, see Section 2.3). The distance between the movable and fixed contacts was as high as *a* = 25 mm. In turn, the ratio of the horizontal and vertical movements of the moving contact, (along the line of the force action), Δ*x* and Δ*z*, respectively, can be determined using the equation:

(1) $$\frac{\Delta x}{\Delta z}\approx \frac{2El}{a\sigma },$$

where *l* = 50 mm is the distance from the axis of rotation of the movable contact to its contact area (Figure 2). Using Equation (1), it is easy to calculate that the horizontal movement of the movable contact exceeds the vertical by more than three orders of magnitude even with *σ_max_*. Therefore, the bending of the sample can be neglected with the selected method of stretching.

The whole setup was controlled by a homemade program that allowed to set the AC frequency range and use the algorithms for changing the magnetic field or temperature and automatically collect the data.

### 2.3. Experiment Conditions

The impedance variations were obtained for the frequency range of an alternating current, *f*, from 0.1 to 100 MHz with an effective current intensity of 1 mA. The external magnetic field, *H*, was oriented along the long side of the ribbon. Its maximum intensity, *H_max_*, was as high as 12 kA/m. The tensile stresses, *σ*, were created by the force acting along the long side of the ribbon. The maximum tensile stress value was 690 MPa. The impedance was measured in the temperature range of 295–405 K. The magnetoimpedance effect ratio was calculated as follows:

(2) $$MI(H)=\frac{Z(H)-Z(H_{max})}{Z(H_{max})}\cdot 100\%,$$

where *Z*(*H*) and *Z* (*H_max_*) are the impedance moduli in the magnetic fields *H* and *H_max_*, respectively. The stress-impedance effect value was determined by the equation:

(3) $$SI(\sigma )=\frac{Z(\sigma )-Z(\sigma =0)}{Z(\sigma =0)}\cdot 100\%$$

where *Z*(*σ*) and *Z*(*σ* = 0) are the impedance moduli at certain tensile stresses *σ* and *σ* = 0 MPa, respectively.

## 3. Results

**Figure 3:**
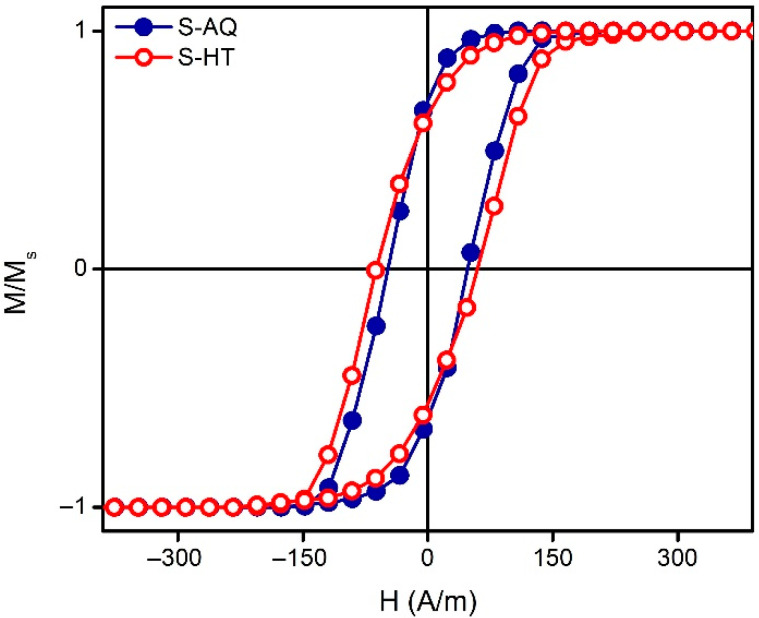
Magnetic hysteresis loops of the as-quenched amorphous ribbons (S-AQ) and the amorphous ribbons after the heat treatment (S-HT). Measurements were made at the temperature of 295 K.

Magnetic hysteresis loops were measured by the induction method in a longitudinal magnetic field with a frequency of 1 kHz. The magnetic field amplitude for these measurements was ±1.5 kA/m. In the as-quenched state, the investigated amorphous ribbons can be described as soft ferromagnets with longitudinal effective anisotropy and a low coercivity of about 50 A/m, The heat treatment of the ribbons leads to a slight increase in the anisotropy field and coercive force (Figure 3). The remnant magnetization, in contrast, slightly decreases after the heat treatment, indicating the existence of some non-uniform stress relaxation processes. It might be due to the difference in the stress relaxation peculiarities of the surface and the volume parts of the ribbon.

### 3.1. MI of the Co_68.5_Fe_4_Si_15_B_12.5_ Ribbons at the T = 295 K before and after Heat Treatment

**Figure 4:**
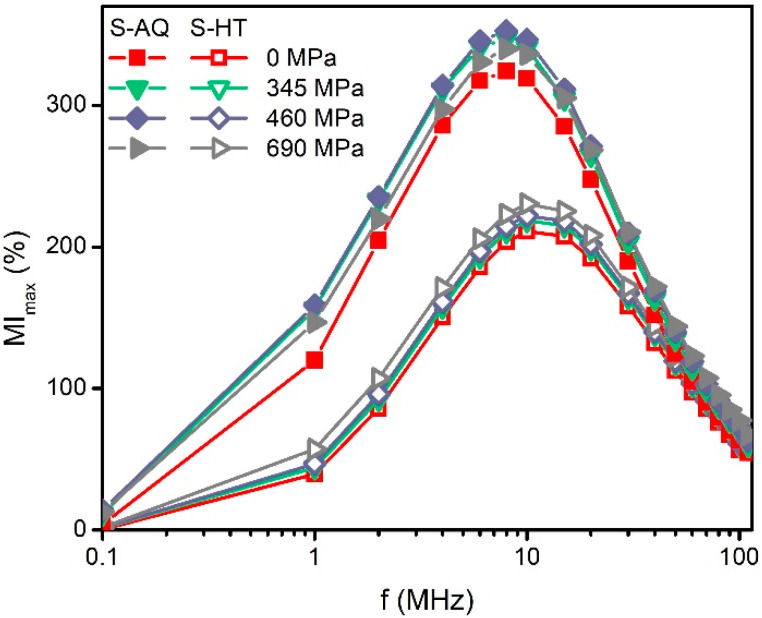
Frequency dependencies of the maximum value of MI, *MI_max_*. Dependencies are given for the following values of the tensile stresses: 0, 345, 460 and 690 MPa. Filled symbols correspond to the S-AQ sample, and the empty symbols correspond to the S-HT one.

**Figure 5:**
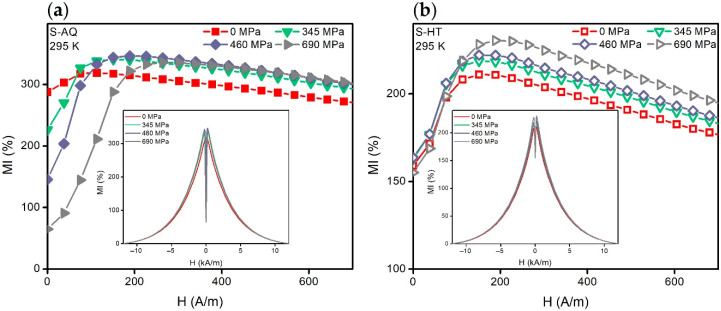
Magnetoimpedance dependencies *MI*(*H*) of the S-AQ (**a**) and the S-HT (**b**) samples. Dependencies were obtained at the AC frequency of 10 MHz at the following tensile stresses: 0, 345, 460, and 690 MPa. The insets show the same *MI*(*H*) dependencies in the *H* range of −12 to 12 kA/m.

Figure 4 shows the dependencies of the maximum magnetoimpedance ratio *MI_max_* on the alternating current frequency value. The value of *MI_max_* corresponds to the maximum of the *MI*(*H*) dependence calculated using Equation (2) (see, for example, Figure 5). It can be seen that the *MI_max_*(*f*) curves of the S-AQ sample have maxima of *f* ≈ 8 MHz at whole mechanical stress (Figure 4, filled symbols). An increase in mechanical stresses in the range of 0–460 MPa causes a noticeable increase in the *MI_max_*. Thus, the increase in the *MI_max_* was close to 30% at a frequency of 8 MHz and it reached the maximum value of 350%. However, the further increase in *σ* lead to a slight decrease in *MI_max_*.

The MI value of the ribbons becomes smaller after the heat treatment (Figure 4, empty symbols). The *MI_max_* decreased more than 100% in the alternating current frequency range of 1–10 MHz. The maxima of the *MI_max_*(*f*) dependencies were observed at the frequency of about 10 MHz. The increase in mechanical stress leads to an increase in *MI_max_*, but it did not exceed 20%.

The thermal reversibility features of the *MI* of the S-HT amorphous ribbons was also investigated. The change in the MI measured at room temperature after heating up to 405 K did not exceed ± 6%, being related to the value measured before such a heating.

For *T* = 295 K, the mechanical stress application caused strong changes in the *MI*(*H*) dependencies of the S-AQ amorphous ribbons, without any significant change of *MI_max_* value (Figure 5a). Thus, when *σ* = 0 MPa, the *MI*(*H*) curve had a weakly pronounced ascending part. This part became much more pronounced with the increase in the mechanical stresses. The field strength, *H_p_*, which was necessary to achieve *MI_max_*, was increased. As *σ* was increasing, *MI*(*H* = 0) decreased and approached the zero value (Figure 5a, insert). The MI sensitivity to the magnetic field in the range of 0 to *H_p_* increased with the mechanical stresses increase from 0 to 575 MPa from 0.4%/(A/m) to 2%/(A/m), but decreased slightly with the further increase in *σ*.

*MI*(*H* = 0) of the S-HT sample did not change very much under the application of the mechanical stresses (the change is less than 8%). *H_p_* also varied insignificantly. However, the ascending part of the *MI*(*H*) curve was increased (Figure 5b). The *MI* sensitivity with respect to the external magnetic field in the range of 0–*H_p_* increased slightly from 0.5 to 0.6%/(A/m) with a mechanical stresses value increase.

In addition, it can be mentioned that the difference between the *MI_max_* values of the ribbons in the as-quenched state and after the heat treatment (for all the values of the applied mechanical stresses) becomes insignificant for the frequencies of the alternating current above 40 MHz.

### 3.2. MI and SI of the Heat-Threated Co_68.5_Fe_4_Si_15_B_12.5_ Ribbons in the Tempearature Range from 295 to 405 K

**Figure 6:**
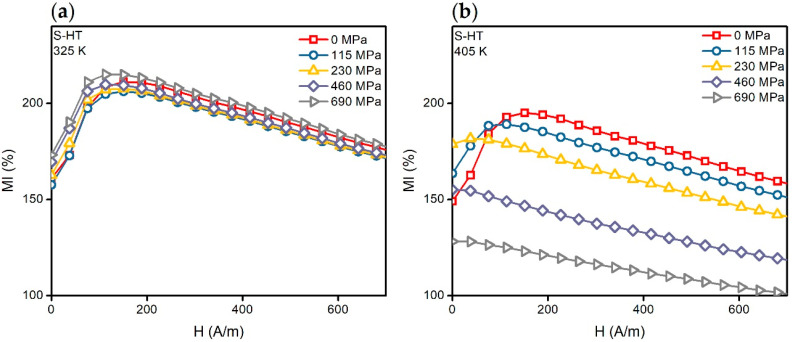
Magnetoimpedance dependencies *MI*(*H*) of the S-HT amorphous ribbon obtained at the temperatures *T* = 325 K (**a**) and *T* = 405 K (**b**). The dependencies are given for the alternating current frequency of 10 MHz and the tensile stresses of 0, 115, 230, 460, and 690 MPa.

**Figure 7:**
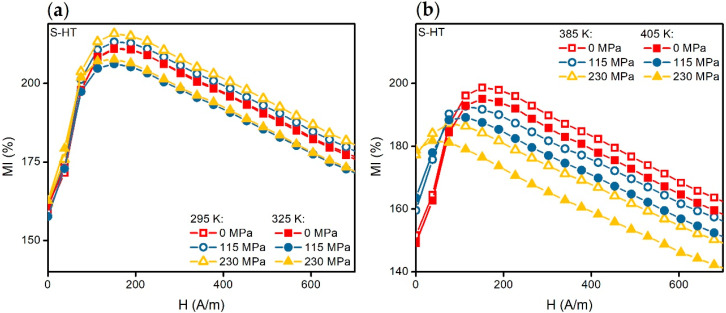
Magnetoimpedance dependencies *MI*(*H*) of the S-HT sample obtained at the temperatures: (**a**) 295 and 325 K; (**b**) 385 and 405 K. The dependencies are given for the alternating current frequency of 10 MHz and the tensile stresses of 0, 115 and 230 MPa.

In the temperature range from 295 to 325 K, the character of the effect of the mechanical stresses on the *MI*(*H*) dependencies of the S-HT type samples did not change (Figure 5b and Figure 6a). It is important to note that the ascending parts of the *MI*(*H*) curves obtained in the temperature range of 295 to 325 K and the mechanical stresses of 0 to 230 MPa practically coincide with each other. However, the MI sensitivity with respect to the external magnetic field in the range of 0–*H_p_* remained almost constant (Figure 7a). It is also worth mentioning that with the higher temperatures, the situation was different (Figure 7b).

The *MI*(*H*) dependencies undergo significant change under the application of mechanical stresses when *T* > 325 K (Figure 6b). Thus, with the *σ* increase, in the beginning the ascending part of the *MI*(*H*) curve becomes less and less pronounced, and then the ascending tendency completely disappears. In the other words, the *H_p_* decreases down to the value of zero.

**Figure 8:**
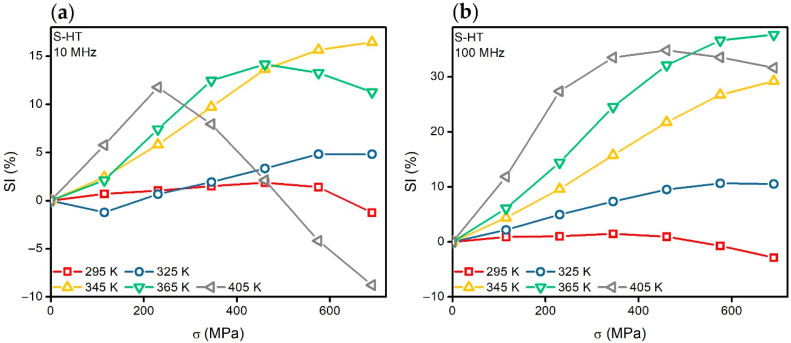
Stress-impedance dependencies *SI*(*σ*) of the S-HT amorphous ribbons. The dependencies were obtained at the alternating current frequencies of 10 MHz (**a**) and 100 MHz (**b**). They are given for the temperatures: 295, 325, 345, 365, and 405 K.

The features of the stress-impedance dependencies *SI*(*σ*) calculated using Equation (3) are also different in the temperature ranges from 295 to 325 K and from 325 to 405 K (Figure 8a). In the temperature range from 295 to 325 K, the change in the impedance under the application of the mechanical stresses did not exceed 10% in the all alternating current frequency range. When the temperature increases above 345 K, the stress-impedance value increases and exceeds 30% at the alternating current frequencies above 40 MHz (Figure 8b).

The mechanical stresses *σ_p_* which were necessary in order to achieve the maximum of the SI value decreased with an increase in the temperature. For example, at *f* = 10 MHz, *σ_p_* decreased from 460 to 230 Mpa, with an increase in the temperature from 365 to 405 K (Figure 8a). Note that under the mechanical stresses close to *σ_p_*, the ascending part of the *MI*(*H*) curves disappeared completely (Figure 6b).

## 4. Discussion

The heat treatment of the Co_68.5_Fe_4_Si_15_B_12.5_ amorphous ribbons at the temperature of 425 K led to a noticeable decrease in the magnitude of the magnetoimpedance effect. However, the magnetic field sensitivity of the MI significantly increased at *σ* = 0 MPa. Good thermal reversibility of the MI was also achieved in the temperature range from 295 to 405 K with no structural transition in the ribbons, and their state was kept amorphous despite some stress relaxation.

Moreover, the MI sensitivity with respect to the magnetic field of the heat-treated ribbons varied very little in the temperature range from 295 to 325 K under the influence of the mechanical stresses (Section 3.1., Figure 7a). We mentioned in the Introduction that in the composite materials the temperature change results in appearance of mechanical stresses in the MI element due to the difference in thermal expansion coefficients of the MI sensor materials. It affects the thermal stability of the MI sensor characteristics. Therefore, the results obtained in the present study can be useful for practical applications. In particular, the temperature range from 295 to 325 K, including normal human body temperature, can be sufficient for the biomedical applications of the materials with such a temperature interval of thermal stability [17,19].

It was reported previously that for the amorphous alloys of similar compositions, heat treatments at temperatures above 375 K cause structural relaxation, affecting the magnetoelastic properties [32,33]. We suppose that the change in the effect of mechanical stresses on the MI of the ribbons after heat treatment (Figure 4) is associated with a change in their magnetostriction.

The impedance module of a ferromagnetic planar conductor of thickness *d* can be represented using the following equation [5,34]:

(4) $$Z=\frac{kR_{DC}}{2\left(\cosh k-\cos k\right)}\;\sqrt{{\left(\sin k+\sinh k\right)}^{2}+{\left(\sin k-\sinh k\right)}^{2}},$$

where *R_DC_* is the DC resistance; *k* = *d*/*δ*; *δ* = (*ρ*/*πfμ*_0_*μ_t_*)^1/2^ is the thickness of the skin layer; *f* is the frequency of the alternating current; *ρ* is the electrical resistivity; *μ_0_* is the magnetic constant; *μ_t_* is the effective transverse (relative to the direction of the alternating current) magnetic permeability. Thus, the temperature changes in Z, and therefore MI (see Equation (2)), will be determined by the temperature changes in the magnetic and electrical properties. Note that the temperature changes in *ρ* and *R_DC_* of soft magnetic alloys are insignificant in comparison with the temperature changes in *μ_t_* [20,35].

**Figure 9:**
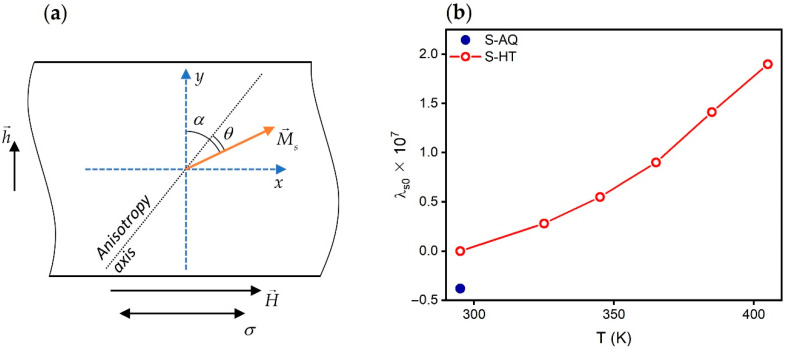
(**a**) Scheme for Equation (5). (**b**) Temperature dependence of the magnetostriction of the S-HT amorphous ribbon (empty symbols). The filled symbol shows the magnetostriction value of the S-AQ amorphous ribbon at room temperature.

Assuming that the magnetization vector and the anisotropy axis lie in the plane of the ribbon, we can write the equation for the free energy functional [36]:

(5) $$W=K\sin^{2}\theta +\frac{3}{2}\lambda_{s}\sigma \cos^{2}(\alpha +\theta )-\mu_{0}M_{s}H\cos(\alpha +\theta )+\mu_{0}M_{s}h\sin(\alpha +\theta ),$$

where *K* is the constant of the effective anisotropy; *λ_s_* is the saturation magnetostriction constant; *h* is the AC field; *α* is the angle between the anisotropy axis of the ribbon and the transverse direction; *θ* is the angle between the axis of anisotropy and magnetization (Figure 9a). Using the standard procedure described, for example, in [23], one can obtain the following equation for transverse magnetic permeability:

(6) $$\mu_{t}=1+\frac{\mu_{0}M_{s}^{2}\sin^{3}(\alpha +\theta )}{3\lambda_{s}\sigma \sin(\alpha +\theta )+\mu_{0}M_{s}H\left(2-\sin^{2}(\alpha +\theta )\right)-2K\sin(\theta -\alpha )}.$$

Thus, the temperature changes in the transverse magnetic permeability will be determined by the temperature changes in the magnetization, effective anisotropy and magnetostriction.

Let us evaluate the influence of the temperature changes in magnetization and effective anisotropy on the MI for the case of S-HT ribbons. Considering the *MI*(*H*) dependencies at *σ* = 0 (Figure 5b and Figure 6), we can see that the field *H_p_* practically does not change with the temperature change. In this case, *H_p_* ≈ *H_K_* [37], where *H_K_* is the effective anisotropy field. Solving the equation *∂W/∂θ* = 0, we can show that:

(7) $$H_{K}∼\frac{2K-3\lambda_{s}\sigma }{M_{s}}.$$

For *σ* = 0, we obtain that *H_K_* ~ *K/Ms*. Thus, taking into account the weak temperature change in *H_p_*, we can conclude that the temperature changes in the magnetization and effective anisotropy do not significantly affect the MI. Most likely, not only the *K/Ms* ratio, but also the values of the *M_s_* and *K* change slightly, since the studied temperatures are much lower than *T_C_*.

The equilibrium magnetization orientation *θ* necessary for *μ_t_* calculating can be determined from the conditions *∂W/∂θ* = 0 and *∂^2^W/∂θ^2^* > 0. For an arbitrary value of *α*, the solution of this problem is possible only by numerical methods [23]. However, for the purposes of our analysis, it suffices to take into account that under the action of the mechanical stresses, the angle *θ* will decrease in the case of the negative magnetostriction, that is, the magnetization will approach the transverse direction, and in the case of a positive one, vice versa [23]. It follows from Equation (6) that this will affect the dependencies *μ_t_*(*H*) and consequently, the dependencies *MI*(*H*) (see Equations (4) and (2)). In the first case, the field *H_p_* ≈ *H_K_* and the ascending part on the *MI*(*H*) dependency will increase, and in the second case, they will decrease [38,39]. It also follows from Equation (6) that the greater the magnetostriction, the more pronounced these changes will be.

Let us turn to the S-AQ magnetoimpedance dependencies obtained at room temperature (Figure 5a). When σ = 0 MPa, *MI*(*H*) has a slightly pronounced ascending part, which indicates an existence of a predominantly longitudinal effective magnetic anisotropy [38,39]. The increase in the ascending part of the *MI*(*H*) and its maximum shift toward the high fields with increasing tensile stresses indicate the negative value of the effective magnetostriction coefficient, as shown above.

The *MI*(*H*) curves of the S-HT amorphous ribbons contain the well defined ascending part at *σ* = 0 MPa. They change very little in the temperature range from 295 to 325 K with increasing tensile stresses (Figure 5b and Figure 6a). This is probably due to the almost zero magnetostriction value. However, one can see significant changes in the magnetoimpedance dependencies under the action of mechanical stresses at *T* > 325 K (Figure 6b). The ascending part becomes less pronounced with an increase in *σ*. It disappears at a certain value of mechanical stresses, *σ_p_*. In turn, the field *H_p_* decreases with the increasing of the mechanical stress and it becomes equal to zero at *σ* ≈ *σ_p_*. Such changes under the action of the tensile mechanical stresses indicate positive magnetostriction. Note that states for which the ascending part disappears in the *MI*(*H*) curve (Figure 6b) correspond to the predominantly longitudinal orientation of the magnetization (even at *H* = 0) [38,39]. We also noted (Section 3.2) that *σ_p_* decreases with a temperature increase, which is presumably due to an increase in magnetostriction.

The magnetostriction values for the S-AQ and S-HT samples were determined from the increment of the field *H_p_* caused by the change in *σ*, under the assumption that the *H_p_* field is close to the effective magnetic anisotropy field [37]. The dependence of the value of the effective magnetostriction coefficient on the mechanical stresses value was also taken into account, which can be expressed as follows [40,41]:

(8) $$\lambda_{s}=\lambda_{s0}-\beta \sigma ,$$

where *λ_s_*_0_ is a magnetostriction value in the absence of the mechanical stresses, *β* is a coefficient usually taking a value in the range of (1 ÷ 6) × 10^−10^ MPa^−1^. As can be seen, the *λ_s_*_0_ of the S-AQ amorphous ribbons at room temperature is negative and is approximately equal to −0.4 × 10^−7^ (Figure 9b, filled symbol). Close magnetostriction values for the ribbons with similar compositions were obtained by other authors [26,37,40,42].

The magnetostriction coefficient for the S-HT amorphous ribbons is positive over the entire studied temperature range. It increases with a temperature increase (Figure 9b, empty symbols). In the temperature range from 295 to 325 K, the value of *λ_s_*_0_ is very small, and it does not exceed 0.3 × 10^−7^.

The near-zero value of magnetostriction around 295 K allows us to suggest that this temperature is the temperature of the magnetostriction compensation for the Co_68.5_Fe_4_Si_15_B_12.5_ heat-treated amorphous ribbons. The presence of the compensation temperature is a characteristic feature for the amorphous CoFeSiB alloys. It is explained by the competition of single-ion and two-ion interactions [43,44]. Even a small content of Fe atoms in an amorphous Co-based alloy makes a significant contribution to the competition of single-ion and two-ion interactions [44].

Considering these results, we can conclude that it is important to achieve near-zero magnetostriction values for the MI element in a wider temperature range if the goal is to expand the temperature ranges with a high thermostability of the MI sensors. As well, the materials of the MI sensor with the thermal expansion coefficient close to that for the MI element should be used. Note that to some extent the magnetostriction of the amorphous alloys and its temperature dependence can be controlled by heat treatment and by varying their compositions [40,41,43,44].

On the other hand, for complex composite materials like multilayered structures, it is possible to select a material of the substrate with a desired temperature expansion coefficient. In this case, the mechanical stresses arising in the MI element could compensate the temperature changes and control the MI. Obviously, the thermal expansion of the substrate should be less than that of the MI element in the case of positive magnetostriction. In the case of negative magnetostriction, the ratio should be the opposite. However, this method requires the careful control of the experimental and fabrication conditions.

## 5. Conclusions

The magnetostriction of the Co_68.5_Fe_4_Si_15_B_12.5_ amorphous ribbons changes its value from −0.4 × 10^−7^ to almost zero after low temperature relaxation heat treatment at 425 K for 8 h. The low positive values of the magnetostriction in the heat-treated ribbons are maintained in the temperature range from 295 to 325 K, and cause small changes in the magnetoimpedance under the influence of temperature and mechanical stresses, as well as the low stress-impedance effect. The increase in the magnetostriction with the temperature leads to the increase in the sensitivity of the magnetoimpedance to mechanical stresses and a sufficiently large stress-impedance effect (above 30%) at the temperatures above 325 K.

It is shown that the combined influence of the temperature and the mechanical stresses should be taken into account when the solving issues for increasing the MI sensors’ thermal stability. This is because the MI sensitive element even for the case of supposedly uniform material can be composed of the parts with different temperature expansion coefficients. Therefore, the temperature changes lead the increase in the mechanical stresses in the MI element, affecting the thermal stability of its characteristics.
